# Evaluating Markers of Immune Tolerance and Angiogenesis in Maternal Blood for an Association with Risk of Pregnancy Loss

**DOI:** 10.3390/jcm10163579

**Published:** 2021-08-14

**Authors:** Michelle A. Wyatt, Sarah C. Baumgarten, Amy L. Weaver, Chelsie C. Van Oort, Bohdana Fedyshyn, Rodrigo Ruano, Chandra C. Shenoy, Elizabeth Ann L. Enninga

**Affiliations:** 1 Department of Obstetrics and Gynecology, Mayo Clinic College of Medicine, Rochester, MN 55905, USA; michelleashleywyatt@gmail.com (M.A.W.); Baumgarten.Sarah@mayo.edu (S.C.B.); Vanoort.Chelsie@mayo.edu (C.C.V.O.); Fedyshyn.Bohdana@mayo.edu (B.F.); rodrigoruano@hotmail.com (R.R.); Shenoy.Chandra@mayo.edu (C.C.S.); 2 Department of Health Sciences Research, Mayo Clinic College of Medicine, Rochester, MN 55905, USA; weaver@mayo.edu; 3 Department of Immunology, Mayo Clinic College of Medicine, Rochester, MN 55905, USA

**Keywords:** miscarriage, pregnancy loss, immunology, vascular endothelial growth factor, galectin-9, interleukin-4

## Abstract

Pregnancy loss affects approximately 20% of couples. The lack of a clear cause complicates half of all miscarriages. Early evidence indicates the maternal immune system and angiogenesis regulation are both key players in implantation success or failure. Therefore, this prospective study recruited women in the first trimester with known viable intrauterine pregnancy and measured blood levels of immune tolerance proteins galectin-9 (Gal-9) and interleukin (IL)-4, and angiogenesis proteins (vascular endothelial growth factors (VEGF) A, C, and D) between 5 and 9 weeks gestation. Plasma concentrations were compared between groups defined based on (a) pregnancy outcome and (b) maternal history of miscarriage, respectively. In total, 56 women were recruited with 10 experiencing a miscarriage or pregnancy loss in the 2nd or 3rd trimester and 11 having a maternal history or miscarriage. VEGF-C was significantly lower among women with a miscarriage or pregnancy loss. Gal-9 and VEGF-A concentrations were decreased in women with a prior miscarriage. Identification of early changes in maternal immune and angiogenic factors during pregnancy may be a tool to improve patient counseling on pregnancy loss risk and future interventions to reduce miscarriage in a subset of women.

## 1. Introduction

A positive pregnancy test can be a momentous occasion in a person’s life; however, in the United States alone, 20% of couples will experience a pregnancy loss, which can lead to long-term physical and psychological distress [1,2]. Women who have a miscarriage report feeling isolated, ashamed, and dissatisfied that their clinical care did not address their emotional well-being [3]. While approximately half of first trimester miscarriages are associated with genetic abnormalities or identifiable uterine factors, the other 50% often lack a clear cause [4,5]. Currently, we rely on clinical symptoms of vaginal bleeding and cramping as well as trending human chorionic gonadotropin (hCG) levels and early pregnancy transvaginal ultrasonography to determine an increased risk of pregnancy loss; however, these methods are often unclear in the early stages of a miscarriage and there is a need for more prognostic indicators for pregnancy viability. 

Pregnancy is a unique period that requires alterations in the immune system to accept a haploidentical fetus. Despite investigation into the fetal and maternal interactions at play during gestation, there is still a limited understanding of these complex interactions [6,7,8,9,10,11,12]. There is increasing evidence of the importance of the immune system in the success or failure of a pregnancy. T cell immunoglobulin and mucin-containing protein 3 (Tim-3), a transmembrane protein expressed on differentiated T-helper 1 cells (Th1) and natural killer (NK) cells that functions as an immune checkpoint, has been found to be critical for suppressing allograft rejection [13,14,15,16,17,18] and, thus, it is hypothesized to play a central role in pregnancy outcomes. Studies have demonstrated that Tim-3 expression on various immune cells is increased early in the first trimester and remains elevated throughout pregnancy [19,20,21,22]. Reduced Tim-3 expression has been identified in mice prone to recurrent miscarriage and, additionally, the blockade of the Tim-3 pathway was associated with increased miscarriage rates [20,22,23].

We have previously shown that early in the first trimester plasma galectin-9 (Gal-9) levels increase in maternal blood and remain elevated throughout pregnancy [24]. Gal-9, a ligand for Tim-3, promotes Th1 cell apoptosis, resulting in the downregulation of cytotoxic activity and the promotion of immunologic tolerance [15,18,25]. Interactions between Tim-3 and Gal-9 have also been shown to suppress NK cell cytotoxicity at the maternal–fetal interface [26]. Women who have a history of miscarriage have reduced Gal-9 plasma concentrations, and the administration of Gal-9 reduced embryo loss in abortion-prone mice [22]. Interleukin-4 (IL-4), a cytokine involved in differentiation of naïve CD4+ cells into a Th2, tolerogenic phenotype, increases Tim-3 expression [21,22]. IL-4 expression increases throughout gestation [27,28]. Gal-9 also increases IL-4 concentrations, and in patients with recurrent miscarriage, IL-4 levels have been shown to be decreased [29,30]. This indicates that these interactions have an important role in pregnancy success in early gestation. 

In addition to the Tim-3/Gal-9 pathway, which regulates immune tolerance at the maternal–fetal interface, angiogenic factors, such as vascular endothelial growth factor (VEGF), have been shown to have a critical role in pregnancy through ensuring vascular integrity, which supports implantation and embryo development [31,32,33,34]. Like Gal-9, VEGF concentrations increase in the decidua and serum during the first trimester and activate VEGF receptors [33,35,36]. Histological differences in VEGF expression in trophoblasts, villi endothelial cells, and decidua have been observed with reduced expression in women with a spontaneous miscarriage compared to women with a viable pregnancy [37,38,39]. 

Based on these prior studies, we hypothesized that immunologic dysregulation and alterations in angiogenesis in early pregnancy may account for some causes of unexplained miscarriage and could be measured through the profiling of maternal blood. This study aimed to determine if early plasma protein concentrations including Gal-9, IL-4, VEGF-A, VEGF-C, and VEGF-D were associated with pregnancy loss. This is critical because, despite miscarriage affecting many women, there is an incomplete understanding about the interaction between maternal immune factors, the haploidentical fetus, and angiogenesis, leaving providers with limited tools to help counsel women on risk for or cause of miscarriage or to prevent pregnancy loss.

## 2. Materials and Methods

### 2.1. Patient Selection

This study was approved by the Mayo Clinic Institutional Review Board and patients were recruited either under IRB 18-011413 (*n* = 63) or 13-008482 (*n* = 3). The latter IRB was part of an initial pilot study. A prospective, observational study recruited 66 women presenting to the obstetrical unit for prenatal care between 5 weeks and 9 weeks gestation. Patients could be presenting for either a scheduled visit or due to a pregnancy-related concern (bleeding, abdominal pain, or worsening anxiety due to prior miscarriages or ectopic pregnancy). Women, 18 years of age or older, were approached if they had a confirmed viable intrauterine pregnancy (fetal pole with cardiac activity within the uterine cavity) and were planning to deliver at our institution or health system to obtain pregnancy outcomes from their medical records. The study excluded women who were non-English speaking or less than 18 years old. Written informed consent was obtained by all participants involved in the study. Participants were asked if they had experienced any bleeding or cramping prior to enrollment and, once enrolled, they had 10 milliliters of maternal blood collected within 24 h of enrollment. Whole blood was processed with plasma separated and stored at −80 °C until use. Maternal history, pregnancy symptoms, and outcomes were obtained from a medical chart review. Study data were recorded and managed using a secure Research Electronic Data Capture system (REDCap) [40].

### 2.2. Enzyme Linked Immunosorbent Assays (ELISA)

The following plasma proteins were measured by ELISA to assess immune tolerance: Gal-9, IL-4, VEGF-A, VEGF-C, and VEGF-D. Sandwich ELISAs were used to quantify each protein as per the manufacturer’s instructions (R&D Systems, Minneapolis, MN, USA). All samples were run in duplicate, averaged, and a seven-point standard curve was used to calculate concentrations following capture of 450 nm absorbance using a plate reader (BioTek Instruments Inc., Winooski, VT, USA). 

### 2.3. Aims

The primary aims were to determine if Gal-9, IL-4, VEGF-A, -C, and -D concentrations differ between women who experience (a) miscarriage versus ongoing pregnancy at 12 weeks gestation or (b) pregnancy loss compared to live birth. The secondary aims were pre-specified to compare concentrations based on maternal history of miscarriage compared to no miscarriage and to determine if concentrations differed among fetal sex. 

### 2.4. Statistical Analysis

Data are descriptively summarized using frequency counts and percentages for categorical variables, means and standard deviations (SD) for non-skewed continuous variables, and medians and interquartile ranges (IQR) for continuous variables with skewed distributions. Comparisons between groups were evaluated using the Mann–Whitney test U test. Additionally, 95% confidence intervals (95% CI) for the median difference between groups were calculated using the Hodges–Lehmann estimation of location shift, also known as Moses confidence limits. Spearman rank tests were utilized to identify correlations between plasma proteins, maternal age, and gestation age at blood draw. All calculated *p*-values were two sided and *p*-values < 0.05 were considered significant. Statistical analysis was performed using SAS version 9.4 (SAS Institute, Cary, NC, USA) and Prism 9.0 (GraphPad, San Diego, CA, USA). 

The study was designed to enroll 70 patients, assuming a miscarriage rate of 15%, with the goal of obtaining data on 10 women with a miscarriage and 60 women with a viable pregnancy at 12 weeks. With this sample size, the study would have 80% power to detect a difference in groups means of 2 standard deviations and 90% power to detect a difference in groups means of 1.16 standard deviations, using a two-sided two-sample *t*-test with a type I error of 5%. However, given the skewed nature of the observed data, the actual data analysis utilized a non-parametric test, the Mann–Whitney U test, instead of the parametric two-sample *t*-test, and therefore, the statistical power of our analysis was 5–10% less than what we projected. 

## 3. Results

In total, 74 women were approached, 66 consented to participate, and 8 declined. After enrollment, it was determined that 10 women never had a confirmed viable intrauterine pregnancy at the time of blood draw and they were excluded, leaving 56 participants. Three women recruited under IRB 13-008482 did not have VEGF-A data.

### 3.1. Patient Characteristics and Pregnancy Outcomes

**Figure 1:**
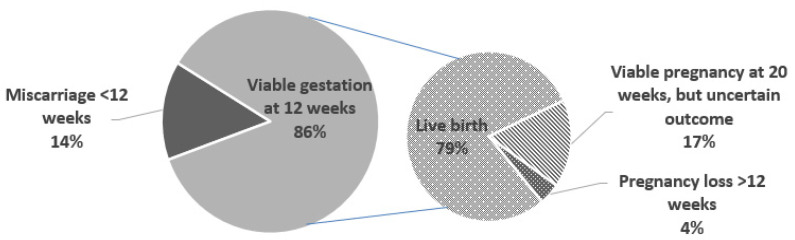
Pregnancy outcomes of the women enrolled on this prospective cohort.

The mean maternal age at recruitment was 30.3 (SD 4.4; range 20–38). The mean gravidity was 2.4 (SD 1.6; range 1–7) and the mean parity was 1 (SD 1.1, range 0–4). Race among participants included 52 (92.9%) self-described as Caucasian, 2 (3.5%) as Asian, 1 (1.8%) as Black, and 1 (1.8%) as other. Table 1 details maternal characteristics based on pregnancy outcomes. Among the 56 included participants, 8 women had spontaneous miscarriage diagnosed prior to 12 weeks gestation, and 2 had losses after 12 weeks gestation. In the loss group, 60% (6/10) of participants had symptoms of bleeding or abdominal pain. In the viable group, approximately a third of women (15/46) had the same concerning symptoms. Genetic testing was not performed in any patient with a pregnancy loss. Overall, 38 women had live births, and 8 women did not have known pregnancy outcomes, but had a viable pregnancy at 20 weeks gestation. (Figure 1) We did not observe strong correlations between the plasma protein concentrations and maternal age or gestational age at recruitment in our study (Supplemental Figure S1). Therefore, we did not fit multivariable models adjusting for these covariates; however, this should be explored in larger studies. 

### 3.2. VEGF-C Is Lower in Women with Miscarriage Prior to 12 Weeks Gestation

Plasma concentrations were compared between the 8 women who experienced a spontaneous loss prior to 12 weeks gestation versus the 48 women with viable pregnancy at 12 weeks. The median concentration of VEGF-C was significantly lower among women who experienced a miscarriage versus those who had an ongoing pregnancy at 12 weeks (1191 pg/mL vs. 2815 pg/mL, respectively, *p* = 0.04). There was no statistically significant difference in median concentrations for Gal-9, IL-4, VEGF-A, and VEGF-D between groups (Table 2).

### 3.3. VEGF-C Is Lower in Women with Pregnancy Loss Compared to Live Birth

**Figure 2:**
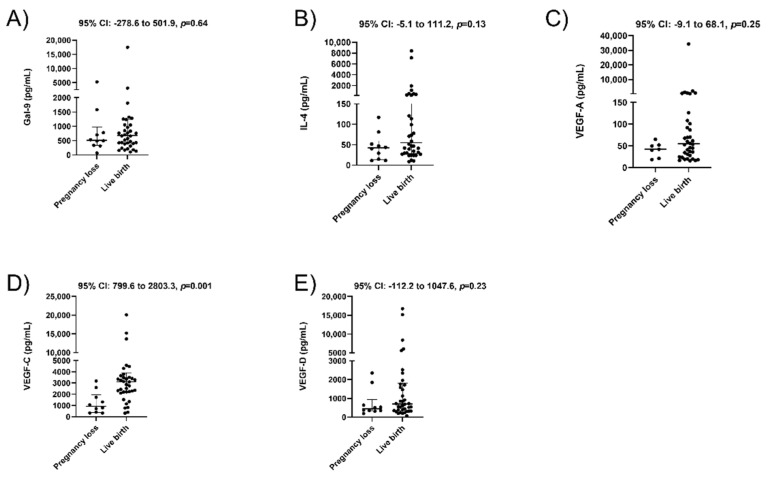
Plasma concentrations of immune tolerance markers and angiogenic factors among women who experience pregnancy loss versus live birth. Concentrations of Galectin-9 (**A**), IL-4 (**B**), VEGF-A (**C**), VEGF-C (**D**), and VEGF-D (**E**) as measured by ELISA. Data are displayed as median with interquartile range along with 95% CI for the median difference between groups above each panel.

We then compared plasma concentrations in 38 women who had a live birth compared to 10 with a pregnancy loss. No significant differences between groups were detected in the concentrations of Gal-9 (Figure 2A, *p* = 0.64) and IL-4 (Figure 2B, *p* = 0.13). VEGF-C levels were again found to be significantly lower among women with pregnancy loss (Figure 2D, median 932.7 vs. 3116.2 pg/mL, *p* = 0.001). Additionally, VEGF-A (Figure 2C, *p* = 0.25) and -D (Figure 2E, *p* = 0.23) concentrations were not significantly different between the two groups. 

### 3.4. History of Miscarriage Associated with Decreased Gal-9 and VEGF-A

**Figure 3:**
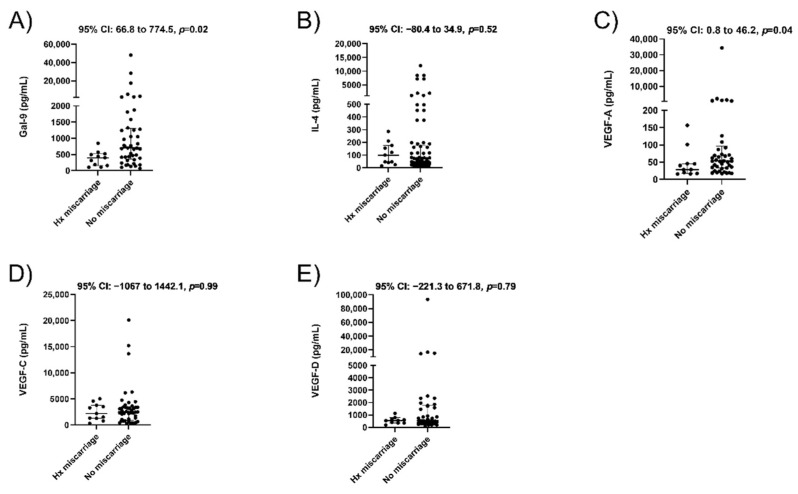
Maternal blood levels of immune tolerance and angiogenesis based on history (Hx) of miscarriage. Concentrations determined by ELISA for Galectin-9 (**A**), IL-4 (**B**), VEGF-A (**C**), VEGF-C (**D**), and VEGF-D (**E**). Data are displayed as the median with interquartile range along with 95% CI for the median difference between groups above each panel.

**Figure 4:**
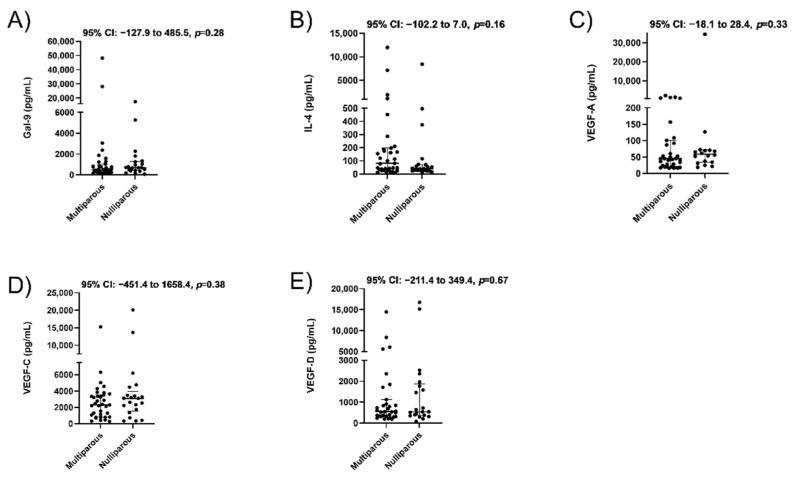
Plasma concentrations in maternal blood from women who are nulligravid (*n* = 21) versus women with prior pregnancy (*n* = 35). Concentrations of Galectin-9 (**A**), IL-4 (**B**), VEGF-A (**C**), VEGF-C (**D**), and VEGF-D (**E**) determined by ELISA. Data are displayed as median with interquartile range, along with 95% CI for the median difference between groups above each panel.

We then wanted to know if a history of miscarriage impacts protein levels in a subsequent pregnancy. Among our cohort, 11 women had a prior spontaneous miscarriage. We observed that their median plasma concentrations were significantly lower for Gal-9 (395.8 vs. 701.5 pg/mL, *p* = 0.02, Figure 3A) and VEGF-A (28.5 vs. 53.4 pg/mL, *p* = 0.04, Figure 3C) compared to the 45 women with no prior miscarriage. There were no significant differences in IL-4 (Figure 3B, *p* = 0.52), VEGF-C (Figure 3D, *p* = 0.99) or VEGF-D (Figure 3E, *p* = 0.79). The difference in Gal-9 levels persisted when comparing 11 women with a history of miscarriage to 24 with a prior pregnancy and no miscarriage history (median, 395.8 vs. 761.0 pg/mL, *p* = 0.053, Table 3). While the VEGF-A concentration was lower, it no longer attained statistical significance when excluding primiparous women (median, 28.5 vs. 51.5 pg/mL, *p* = 0.08). No significant differences were seen in maternal plasma concentrations of all proteins in women for which this was their first pregnancy (N = 21) compared to women with a prior positive pregnancy test (N = 35, Figure 4A–E).

### 3.5. No Differences between Male and Female Fetuses

**Figure 5:**
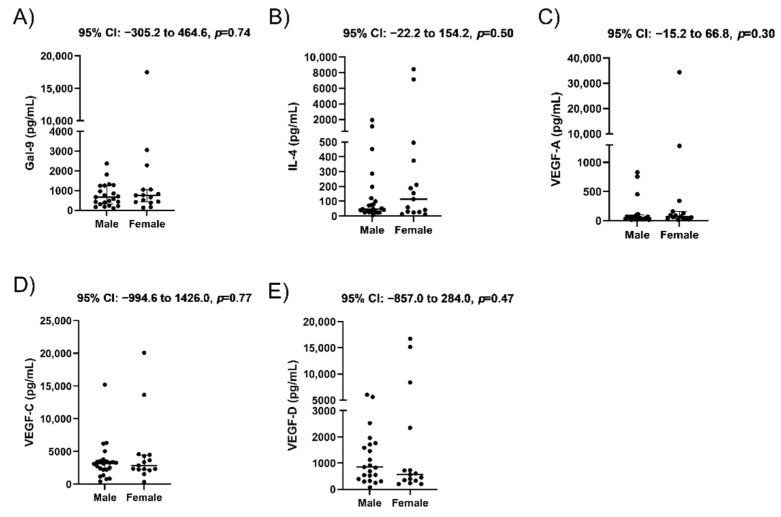
Maternal plasma concentrations based on fetal sex. Concentrations determined by ELISA for Galectin-9 (**A**), IL-4 (**B**), VEGF-A (**C**), VEGF-C (**D**), and VEGF-D (**E**) compared based on the sex of the fetus. Data are displayed as the median with interquartile range, along with 95% CI for the median difference between groups above each panel.

Lastly, we looked for differences in maternal blood protein concentrations by fetal sex among women with a live birth, as we had previously identified differences in maternal blood cytokines and angiogenic factors based on fetal sex [41]. We observed no significant differences between groups (*n* = 15 with a female live birth vs. *n* = 23 with a male live birth) in the concentrations for Gal-9 (*p* = 0.74) or IL-4 (*p* = 0.50) in maternal blood (Figure 5A,B). Additionally, there were no differences between VEGF-A (Figure 5C, *p* = 0.30), VEGF-C (Figure 5D, *p* = 0.77), or VEGF-D (Figure 5E, *p* = 0.47). Therefore, we suspect that fetal sex does not change levels of these cytokines and angiogenic factors in early pregnancy.

## 4. Discussion

The immune system is complex and composed of both innate and adaptive immunity, which protects against foreign antigens (i.e., non-self or pathogens). Gal-9 was selected for assessment of prognostic value based on previous studies that have demonstrated significantly reduced plasma and decidual Gal-9 concentrations among women experiencing a miscarriage compared to a viable pregnancy [22,28]. In the present study, we did not identify significantly lower concentrations of Gal-9 based on the current pregnancy outcomes (miscarriage or live birth). Reasons that our results differed from prior studies may include that prior studies did not report on the gestational age when the samples were collected or the timing in relation to the diagnosis of miscarriage. Additionally, they recruited women with spontaneous miscarriage or undergoing elective termination and, thus, the true pregnancy outcome is unknown. In the present study, Gal-9 was lower in women with a history of pregnancy loss, but not different based on whether this was a women’s first pregnancy. This adds to the hypothesis that Gal-9 levels appear to have a role in pregnancy viability; however, its utility to identify increased risk for adverse pregnancy outcomes needs further assessment. 

Cytokine expression profiles in the decidua appear to favor those produced by Th2 cells, including IL-4, which appears to be partially regulated by Gal-9 [10]. Reduced concentrations of IL-4 in the decidua of women experiencing a recurrent pregnancy loss compared to women with viable pregnancy choosing elective termination have previously been shown [42]; however, there have been limited previous evaluations of plasma cytokine profiles and pregnancy outcomes, which prompted our investigation. The present study did not show any difference in plasma IL-4 concentrations based on maternal pregnancy history nor current pregnancy outcomes. This finding is similar to the work by Marzi et al., which showed peripheral blood mononuclear cells (PBMC) production of IL-4 was not different in women with live births compared to women with a miscarriage or birth of a small for gestational age fetus; however, they obtained samples throughout all gestations of pregnancy [27]. Our findings differed from other studies that showed plasma IL-4 concentrations in early pregnancy were reduced in women with recurrent miscarriages compared to normal pregnancy, but levels measured in the miscarriage cohort occurred at the time of miscarriage diagnosis and not prospectively [22,28]. Based on the findings in our study, maternal blood IL-4 concentrations, obtained early in gestation, do not appear to be associated with the outcome of a pregnancy. 

Angiogenesis is also of great importance during implantation and maintenance of pregnancy. VEGF and its receptors have been identified as key players in normal endometrial vascular remodeling and placentation in pregnancy, with increasing VEGF concentrations throughout the first trimester [36,39,43]. The current study demonstrated that lower blood concentrations of VEGF-C were seen in women who experienced either a miscarriage or pregnancy loss. This suggests that VEGF-C blood levels early in pregnancy may help identify pregnancies at higher risk for adverse outcome, but not be able to predict the timing of event. VEGF-A concentration was significantly decreased among women who experienced a prior miscarriage compared to women without a miscarriage history. These findings support prior work that also found serum levels of VEGF-A and -C obtained outside of pregnancy are decreased in women with a history of recurrent miscarriage compared to women with prior pregnancy and no miscarriage [43,44]. Additionally, this correlates with lower levels of VEGF expression in the decidua and chorionic villi in the first trimester seen in women who experienced recurrent pregnancy loss compared to elective termination [37,39]. These differences in VEGF concentrations highlight their important role in implantation and placental development. 

The current study suggests VEGF plasma concentrations obtained in early pregnancy may be used to identify pregnancies at increased risk of miscarriage or fetal loss and provide a source for intervention. Scarpellini et al. demonstrated that in women with recurrent pregnancy loss, the administration of granulocyte colony stimulating factor (G-CSF) is associated with increased VEGF expression in trophoblasts; however, pregnant women between 8 and 12 weeks gestation without recurrent pregnancy loss and who did not receive G-CSF had higher expression levels of VEGF [45]. This study of G-CSF exposure and our current findings demonstrates the potential to identify interventions or therapies that improve the chance for successful pregnancies once better biomarkers and risk factors for miscarriage are understood. 

In the present study, we also sought to explore if fetal sex altered maternal cytokine secretion, given exposure to a foreign Y chromosome, which may require an additional secretion of immune-suppressing proteins. We previously showed that mothers of male fetuses had increased plasma concentrations of VEGF-A and proinflammatory cytokines throughout pregnancy, although no difference in VEGF-D concentrations were seen between male and female offspring [41]. Placental expression for genes involved in the immune system and graft-versus-host interactions have also shown to be increased in the placentas of male compared to female fetuses born prematurely [46]. In the present study, however, we did not detect a difference in VEGF-A, -C, and -D blood concentrations. Additionally, neither Gal-9 nor IL-4 showed different expression levels based on fetal sex. Our sample was limited to only those with a live birth as genetic information to confirm the fetal sex was not present for any of the pregnancy losses. The lack of significant differences in angiogenic and immune tolerance proteins was an unexpected finding. Further evaluation of factors that do differ between mothers carrying male versus female offspring may allow for a deeper understanding of the interplay of the immune system, angiogenesis, and pregnancy viability. 

While this study did meet the sample size for pregnancy losses required by our power calculation, it is still limited by the small numbers in the setting of a wider range of concentrations than anticipated. Additionally, samples were obtained during a 5-week range in the first trimester. Prior studies have shown plasma concentrations of proteins of interest in the study increase in pregnancy, but there is not strong evidence regarding the gestational age at which they plateau [24,27,28,35]. While there are known factors for pregnancy loss, this study was not able to include genetic information, assessment for uterine factors associated with increased miscarriage risk, or biochemical loss. Repeating the study with a pre-pregnancy measurement and then weekly measurements from 5 until 10 weeks gestation, with genetic testing in all pregnancies, would be of interest to better determine if a certain time point or pattern could be utilized to predict pregnancy outcomes and offer opportunities for intervention in those with unexplained losses. We did not observe meaningful correlations between the concentration levels and maternal age or gestational age at recruitment in our study. Therefore, we did not fit multivariable models adjusting for these covariates; however, this should be explored in larger studies especially if there is more heterogeneity in the maternal age and gestational age at recruitment. Lastly, there is a lack of racial and ethnic diversity in the population. A strength of this study, however, was collecting samples prospectively in women with confirmed intrauterine pregnancy and following the pregnancy in its entirety. Many previous prospective studies of pregnant women recruited patients at the time of miscarriage diagnosis or women undergoing elective termination, which could be responsible for differences in our study compared to others.

## 5. Conclusions

Successful pregnancy implantation and growth is complex. While chromosomal factors are known to be critical for pregnancy success, evidence suggests that angiogenic and immune marker expression also has a significant impact on the success or failure of a pregnancy. Among women with a viable early pregnancy, we found that reduced concentrations of Gal-9 and VEGF-A are more common among women with a prior miscarriage, while decreased VEGF-C expression in the first trimester suggested an increased risk of miscarriage or fetal loss. Further understanding and identification of altered biomarkers prior to or early in pregnancy that increase miscarriage risk is important. Developing thresholds and predictability of serum levels could lead to better pregnancy counseling and the development of potential therapeutic strategies to prevent pregnancy loss.

## Figures and Tables

**Table 1:** Maternal and obstetric characteristics in women with pregnancy loss compared to women who had live birth or viable pregnancy at 20 weeks gestation.

Characteristic	Miscarriage or Pregnancy Loss (*n* = 10)	Viable Pregnancy or Live Birth (*n* = 46)
Maternal Age, mean (SD)	31.7 (3.7)	30.0 (4.6)
Body mass index pre-pregnancy, mean (SD)	30.3 (6.3)	28.2 (7.0)
Gravidity, mean (SD)	2.1 (1.8)	2.5 (1.5)
Parity, mean (SD)	0.9 (1.5)	1.0 (1.0)
Prior spontaneous miscarriage, N (%)	1 (10%)	10 (22%)
Race, N (%)		
Caucasian	9 (90%)	43 (94%)
Black	1 (10%)	0 (0%)
Asian	0 (0%)	2 (4%)
Other	0 (0%)	1 (2%)
Maternal comorbidities, N (%)		
Pregestational diabetes	0 (0%)	3 (6.5%)
Chronic hypertension	2 (20%)	0 (0%)
Thyroid disorder	0 (0%)	2 (4.3%)
Rheumatologic condition	0 (0%)	1 (2.2%)
Gestational age at recruitment, N (%)		
5 weeks	1 (10%)	3 (6.5%)
6 weeks	5 (50%)	10 (21.7%)
7 weeks	2 (20%)	6 (13.0%)
8 weeks	2 (20%)	18 (39.1%)
9 weeks	0 (0%)	9 (19.6%)
Symptoms, N (%)		
Yes	6 (60%)	15 (32.6%)
No	4 (40%)	31 (67.4%)

**Table 2:** Plasma concentrations for immune tolerance markers and angiogenic factors based on pregnancy outcome at 12 weeks gestation.

Plasma Concentrations (pg/mL)	Miscarriage (*n* = 8) Median (IQR)	Viable Gestation (*n* = 48) Median (IQR)	95% CI for the Median Difference between Groups †	*p*-Value
Gal-9	513.2 (396.9, 1179.9)	662.0 (273.6, 1145.7)	−375.8 to 461.3	0.99
IL-4	36.6 (13.2, 64.1)	55.2 (30.3, 192.7)	−2.6 to 143.6	0.08
VEGF-A *	46.2 (40.2, 52.1)	52.8 (24.8, 100.9)	−19.9 to 51.8	0.65
VEGF-C	1191.0 (607.8, 2172.6)	2815.1 (1320.5, 3718.3)	80.9 to 2536.7	0.04
VEGF-D	442.2 (349.3, 1193.5)	583.2 (338.4, 1647.1)	−163.4 to 595.3	0.44

† Difference calculated as viable gestation group minus miscarriage group. * Three patients had missing VEGF-A data, two had a miscarriage prior to 12 weeks, and 1 had viable gestation.

**Table 3:** Plasma concentrations in maternal blood from women with a history of miscarriage versus history of prior pregnancy with no history of miscarriage.

Plasma Concentrations (pg/mL)	History of Miscarriage (*n* = 11) Median (IQR)	History of Prior Pregnancy and No Miscarriage (*n* = 24) Median (IQR)	95% CI for the Median Difference between Groups †	*p*-Value
Gal-9	395.8 (156.4, 518.4)	761.0 (272.1, 1430.4)	−2.9 to 937.4	0.053
IL-4	99.3 (41.2, 176.0)	79.7 (28.3, 324.5)	−69.9 to 146.7	0.96
VEGF-A *	28.5 (17.0, 45.8)	51.5 (28.6, 280.5)	−2.9 to 71.7	0.08
VEGF-C	2205.6 (1293.3, 3790.2)	2327.3 (815.4, 3352.7)	−1365.4 to 1153.1	0.71
VEGF-D	548.5 (349.6, 788.9)	543.2 (314.2, 1780.6)	−256.3 to 582.0	0.90

† Difference calculated as history of prior pregnancy and no miscarriage group minus history of miscarriage group. * 3 patients had missing VEGF-A data; 2 with miscarriage prior to 12 weeks and 1 with viable gestation.

## Data Availability

De-identified data are available upon reasonable request by contacting the corresponding author.

## Supplementary Materials

The following are available online at https://www.mdpi.com/article/10.3390/jcm10163579/s1, Supplemental Figure S1: Correlations between plasma proteins, gestational age at recruitment, and maternal age. (A) Correlation between plasma proteins and maternal age. (B) Correlation between plasma proteins and gestational age at blood collection. Spearman rank coefficients are presented. All *p*-values were >0.05.
