# Supplementary figures and images for: Evaluating Markers of Immune Tolerance and Angiogenesis in Maternal Blood for an Association with Risk of Pregnancy Loss
Source: J Clin Med. 2021 Aug 14;10(16):3579. doi: 10.3390/jcm10163579 (PMC8397206; doi:10.3390/jcm10163579)

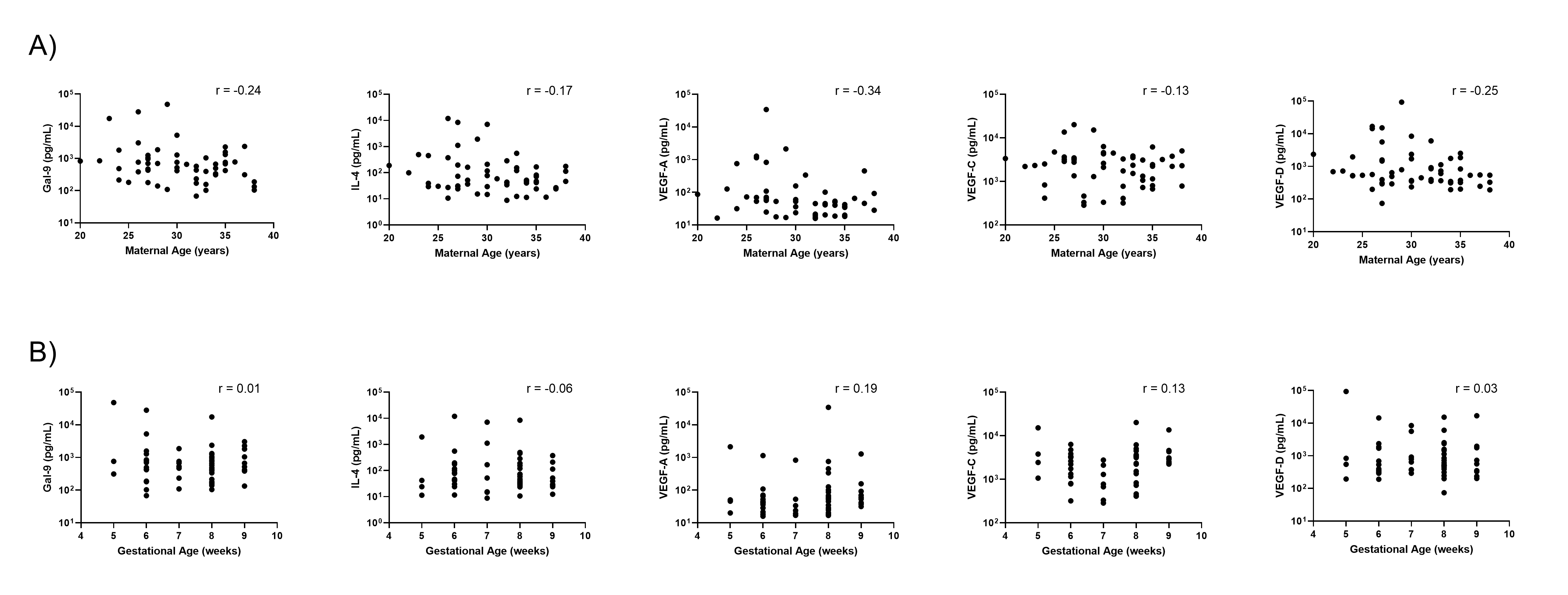

Supplement: Supplementary file 1 [file jcm-10-03579-s001.zip › jcm-1318986-supplementary.tif]
